# Personality Research in Mammalian Farm Animals: Concepts, Measures, and Relationship to Welfare

**DOI:** 10.3389/fvets.2018.00131

**Published:** 2018-06-28

**Authors:** Marie-Antonine Finkemeier, Jan Langbein, Birger Puppe

**Affiliations:** ^1^Institute of Behavioural Physiology, Leibniz Institute for Farm Animal Biology (FBN), Dummerstorf, Germany; ^2^Behavioural Sciences, Faculty of Agricultural and Environmental Sciences, University of Rostock, Rostock, Germany

**Keywords:** personality, temperament, coping style, welfare, farm animals

## Abstract

Measuring and understanding personality in animals is a rising scientific field. Much research has been conducted to assess distinctive individual differences in behavior in a large number of species in the past few decades, and increasing numbers of studies include farm animals. Nevertheless, the terminology and definitions used in this broad scientific field are often confusing because different concepts and methods are used to explain often synonymously applied terms, such as personality, temperament and coping style. In the present review we give a comprehensive overview of the concepts and terms currently used in animal personality research and critically reveal how they are defined and what they measure. First, we shortly introduce concepts describing human personality and how these concepts are used to explain animal personality. Second, we present which concepts, methods and measures are applied in farm animal personality research and show that the terminology used seems to be somehow species-related. Finally, we discuss some findings on the possible impact of personality on the welfare of farm animals. The assessment of personality in farm animals is of growing scientific and practical interest. Differences in theoretical frameworks and methodological approaches may also entail the diverse use of the different concepts between basic and applied research approaches. We conclude that more consistency is needed in using different theoretical concepts, terms and measures, especially in farm animal personality research. The terms coping style and temperament, which are used in different ways, should not be examined as independent concepts, but rather should be considered as different aspects of the whole personality concept. Farm animal personality should be increasingly considered for the improvement of animal housing, management, breeding and welfare.

## Introduction

Measuring animal personality has become quite important in the past few decades, but many studies use different concepts and terminology to explain individual differences in behavior and physiology. Nevertheless, studies on farm animals do measure personality traits with methods that have been used to assess animal behavior. It must be born in mind that the concepts and terms such as personality, temperament, coping style used in animal personality research have their origin in human personality research.

In human personality research, the term personality is used to describe a distinctive and relatively stable set of mental traits that explain the organism's behavior, and the concept of personality is explained with the “Five-Factor Model of Personality.” The five factors describing human personality are extraversion, agreeableness, conscientiousness, neuroticism, and openness (1). Goldberg (2) describes which traits define each factor—extraversion (or surgency) comprises traits like talkativeness, assertiveness and activity levels on one end of the spectrum and silence, passivity and reserve on the other end; agreeableness (or pleasantness) includes traits like kindness, trust and warmth and the opposite traits of hostility, selfishness and distrust; conscientiousness (or dependability) comprises traits like organization, thoroughness and reliability on one end of the spectrum and carelessness, negligence and unreliability on the other end; neuroticism (or emotional stability) includes traits like nervousness, moodiness and temperamentality; and openness to experience (or intellect) describes traits like imagination, curiosity and creativity on one end of the spectrum and shallowness and imperceptiveness on the other end.

The term temperament in human psychology research is closely related to personality. Temperament is defined as the inherited, early appearing tendencies that continue throughout life and serve as the foundation for personality (3–5) because temperament is observable in infants and is tied to basic psychological processes (6, 7). According to Buss (8), the term temperament is used to reflect genetic behavioral differences, while the term personality, which is based on temperament, seems to be used to reflect non-genetic differences. Rothbart et al. (7) suggested that understanding temperament is central to understanding personality because it influences and is influenced by the experiences of each individual and because one of its outcomes is the adult personality. However, most theorists assume that temperament provides the starting place for personality development [reviewed by McCrae et al. (6)]. Temperament provides process-oriented models by establishing links between individual differences in behavior and their psychological and biological substrates in humans as well as in non-human animals, while personality provides subject-oriented models (7).

Coping is a very broad and a multidimensional concept with a long and complex history. In human psychology research, coping strategies refer to intentional cognitive or behavioral attempts by the individual to manage a stressor (9). Lazarus (10) has emphasized coping as a key concept for research on adaptation and health. Because coping is a behavioral reaction to aversive situations that induce several physiological stress reactions (11), individual ways of dealing with stress have an enormous impact on human health (12). Lazarus (10) suggests to differentiating between two approaches to coping, one that treats coping as a personality characteristic (style) and another that refers to the efforts to manage stress (process). Whereas the process approach primarily addresses the behavioral and physiological mechanisms involved in the adaptational response to stress, coping style rather seems to describe a personal or individual consistency in this response. The classification of coping into an “approach-avoidance” category can also be found in animal personality research. This category classifies behavior and cognitive abilities that are used by the individual to face the problem (approach) or to divert the individual's attention away (avoidance) from the problem (13).

Aim of the present review was to give a comprehensive overview how these concepts and terms, which have their origin in human psychology, are currently used in animal and especially farm animal personality research and to critically reveal how they are defined and what they measure. To obtain an overview of published studies in selected mammalian farm animal species we carried out a systematic literature search using the Web of Science (state December 2017) with keywords including the respective animal species (cattle, goat, sheep, horse, pig) and different synonyms applied in personality research (temperament, personality, coping). Then we exemplarily assessed the found studies within the context of terms and methodological approaches in general personality research. Finally, we discuss some findings on the possible impact of personality on the welfare of farm animals to especially highlight a future direction of personality research.

## General concepts and terms in animal personality research

### Personality

In animal personality research, many terms are used to explain individual differences in behavior, besides personality itself [e.g., behavioral syndromes (14) and coping style (15, 16)]. Definitions of the different terms used in animal personality research are shown in Table 1: Personality is defined as a correlated set of individual behavioral and physiological traits that are consistent over time and contexts (15, 29).

**Table 1:** Different personality-related terms in alphabetical order used to describe individual differences in animal behavior and their definitions.

**Personality-related term**	**Definition**	**References**
Behavioral response	Behavioral response to handling	(17)
Behavioral syndrome	A suite of correlated behaviors reflecting an individual's consistency in behavior across multiple situations; a population or species can exhibit a behavioral syndrome; within the syndrome, individuals have a behavioral type	(14)
Coping personality type	Coping strategies that may reflect different personality types	(18)
Coping style	Based on the animal's reaction to its environment with respect to reducing effects of aversive stimuli: - fight or flight response - approach or avoidance - boldness or shyness	(11, 15, 16, 19)
Emotional reactivity	Social reactivity (i.e., active vs. passive strategy); exploratory activity; reactivity to humans	(20)
Identity profile	Describes individuality, personality and their relationship with certain morphological traits of the animals; four groups of similar animals: aggressive, affiliative, passive, avoiders	(21)
Individual differences in behavior	Individual variation; intra-animal repeatability; the relationships between different test situations and the frequency distributions of various measures of behavior; consistency of individual variability	(22–25)
Temperament	Inherited, early appearing tendencies that continue throughout life and serve as the foundation for personality; observable in infants and animals and tied to basic psychological processes	(3–6)
Personality	A correlated set of individual behavioral and physiological traits that are consistent over time and contexts	(15, 26–28)

**Figure 1:**
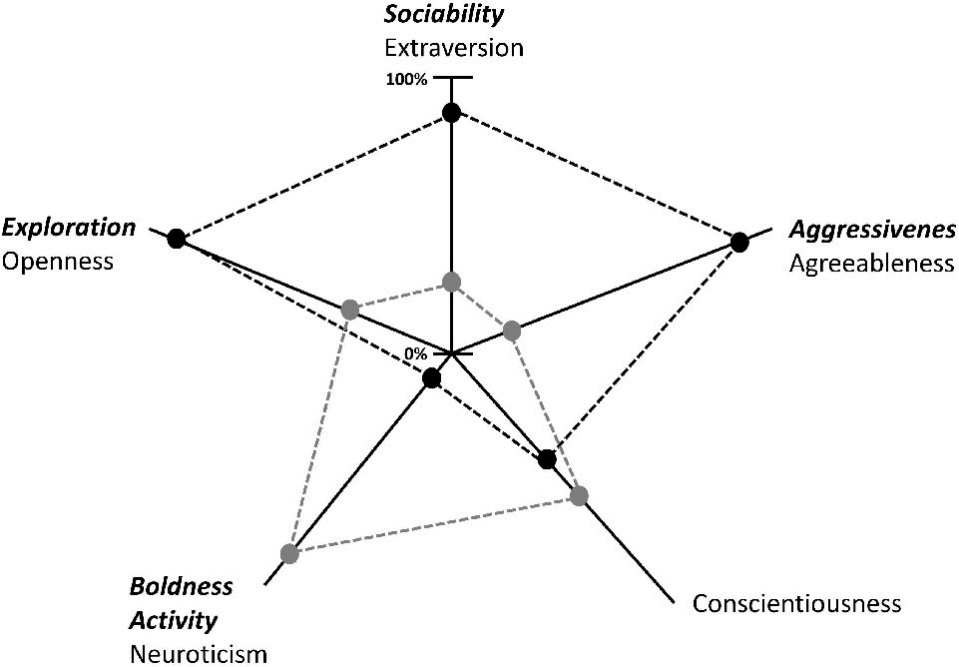
The five personality factors in humans and how they describe the personality type of two imaginary individuals [adapted from (33)]. The equivalent personality factors described in animals are written in bold and italics. Scores are given from zero to 100 in percentages. For example, Individual A (black dashed lines and points) scored high in openness, extraversion and agreeableness, while Individual B (gray dashed lines and points) scored high on neuroticism and conscientiousness.

Personality may explain individual differences in dominance rank, coping, cognitive abilities and physiology. At least one of the five factors which are used to describe human personality (i.e., extraversion, neuroticism, agreeableness, openness, and conscientiousness) has been investigated in many different species. The most commonly measured personality factors in animal personality research are exploration, activity, aggressiveness, sociability, and boldness (29–31). Exploration seems to resemble the human factor openness, aggressiveness seems to resemble agreeableness, and sociability seems to resemble extraversion, while boldness and activity seem to be combined in the human factor neuroticism [reviewed by Gosling and John (32)]. According to Gosling and John (32), the human factor conscientiousness can only be found in chimpanzees and gorillas. Figure 1 shows the five personality factors in humans and how they describe the personality type of two imaginary individuals [adapted from (33)] and the equivalent personality factors found in animals. While individual A scores highly in exploration (openness), sociability (extraversion) and aggressiveness (agreeableness), individual B scores highly on boldness and activity (neuroticism) and, if this individual is a human, in conscientiousness (Figure 1). A discussed factor in animals is the dominance factor that seems to be defined by boldness, physical aggression and low levels of fearfulness (32). In humans, these traits seem to be related to extraversion because humans participate in multiple dominance hierarchies that are less clearly defined and involve widely divergent skills (32). This means that species not only differ in the type of relevant factors, but also in the number of such factors (34): While chimpanzees share a common six-factor structure, including dominance, extraversion, agreeableness, conscientiousness, neuroticism, and openness (35), mountain gorillas seem to have only four personality factors: dominance, openness, sociability and proto-agreeableness (36). As in human personality research, sex differences can be found in the expression of animal personality traits. For example, hyena (*Crocuta crocuta*) males are more fearful and nervous than females. This effect seems to be influenced by the social organization. In hyenas, the dominance rank is transmitted through a matrilineal system, and females are larger than males. Sex differences in personality seem to be related to the ecological niches occupied by the two sexes [reviewed by Gosling and John (32)].

David and Dall (37) state in their review that most of the confusion about terminology in animal personality studies is due to two main concepts, which can also be used complementarily: the intra-individual variability and the life-history approach. The first concept tries to explain the occurrence of between-individual differences in the consistency of any behavior (34), which means that studies employing this concept focus on the consistency and flexibility of behavioral expression. The second concept focuses only on boldness-like behaviors, which means that studies using this concept concentrate on the individual average behavior and the between-individual relationships between life history and behavior (37). One approach using both concepts is the pace-of-life syndrome (POLS) [reviewed by Réale et al. (26)]. This concept includes the idea that between-individual differences in behavior are associated with individual variations in life-history traits [e.g., habitat use, predation avoidance, dispersal and/or social behavior (14, 38–40)] because both may contribute to evolutionary trade-offs and may have co-evolved. And physiological traits that differ between individuals, such as growth, metabolic rate, reproductive value and hormone levels have also become important for animal personality research (28, 40–42) because they can be influenced by genetic predispositions and early environmental effects (27, 43).

Another important aspect of personality research in animals is that despite the consistency of some factors, others seem to be plastic and help the organism adapt to changing environments. This aspect is termed behavioral and/or physiological plasticity (43). Responsiveness to environmental conditions may span from short-term (phenotypic flexibility) to long-term effects (44), and the early environment an individual experiences contributes to between-individual differences in life histories and personality factors (41, 45). For example, in coral reef fish, individual scores on activity, boldness and aggressiveness increased from 2.5- to 6-fold when induced by an increase in water temperature, indicating individual coping abilities and/or behavioral plasticity (46). Moreover, photoperiodic changes influenced fearlessness, the stress response, the timing of maturation and resting metabolic rate (RMR) in wild cavies (*Cavia aperea*) and showed differences between sexes (47, 48). Behavioral and physiological plasticity is an important factor for the survival of an organism. Even if most of the personality factors proved to be consistent over time and contexts, factors like RMR and fearlessness were not consistent in cavies, whereas exploration and boldness were consistent regardless of the photoperiodic treatment (47). One might argue that factors such as RMR and fearlessness should not count as personality factors. However, an example in the European mink (*Mustela lutreola*) showed that traits that describe factors like boldness and exploration can change between seasons but that these changes remain consistent over time (49), which matches the definition of a personality factor. These examples indicate that the plasticity and consistency of personality factors are not exact opposites and indicate interesting asymmetries in the adjustments of personality factors that seem somehow species-related.

In farm animal studies, the term personality is scarcely used, even if the methods employed (e.g., repeated measurements) and measured variables (e.g., curiosity) can be described as personality factors. In those studies, the term temperament is used as an equivalent to the term personality. This could be due to the fact that farm animals are used to get meat, milk and other products and that it is easier to consume farm animal products when scientists do not attribute human-like characteristics to explain individual differences in farm animal behavior. Table 2 lists selected studies assessing personality factors using different experimental designs in mammalian farm animals (Table 2A: cattle; Table 2B: goats; Table 2C: sheep; Table 2D: horses; Table 2E: pigs) and the behavioral tests used. Some of these studies only measured and/or analyzed a small aspect of personality, measured just one or two personality traits or are examples of the very beginning of farm animal personality research, while other studies, shown in this table, show consistency and/or cross-context correlations between traits. One can see that the use of a certain personality-related term is dependent on the species studied. In cattle, the term temperament is mostly used to explain individual differences in behavior, while in pigs, the term coping style is mainly used (Tables 2A,E). In horses and more recent farm animal personality studies, one can find the term personality as well [e.g., (18, 76)] (Table 2D). In most of the farm animal studies investigating temperament, different personality factors can be found [e.g., curiosity shown in the general response to unfamiliar humans or the exit from a restraint device (50, 100)]. These can be considered as personality factors when they are measured in at least two different time periods of an animals' life. Like in other taxa, some farm animal studies can show that the personality factor boldness is related to other investigated behavioral traits. Boldness was found to be related to reactivity to humans in horses (77), cattle (50), and sheep (69): Individuals that interacted more with a novel stimulus were more interested in novel humans as well. In sheep, bold individuals proved to be more explorative and the distance between individuals while grazing was greater than in shy individuals (69). Moreover, the personality factor activity proved to be related to boldness and reactivity to humans in sheep: More active sheep seem to be bolder und more reactive to humans than shy sheep (65, 101). Calves can be categorized in four different personality types based on a combination of behavioral and heart rate variability (HRV) data (50).

**Table 2:** Selected studies assessing personality factors using different experimental designs in mammalian farm animals [2 **(A)**: cattle; 2 **(B)**: goats; 2 **(C)**: sheep; 2 **(D)**: horses; 2 **(E)**: pigs].

**Personality-related term used**	**Test**	**Measures**	**Study**
**(A): CATTLE (*****Bos taurus*****)**			
Behavioral response	Docility test	Aggressiveness against the handler, running time and number of escapes per minute of test period in presence or absence of the handler	(17)
Individual differences in behavior	Human approach test Lateralization test Novel human test Novel object test Open field test Surprise test Temperament score	Behavioral response when a human approaches the cow The side the animal uses to avoid an obstacle Behavioral response toward an unknown human Behavioral response toward an unknown object Activity and exploration behavior in an unknown arena Latency to start eating again after encountering a blast of air Chute score, velocity exit, pen score, flight speed	(23, 24)
Temperament	Combined social isolation and open field test Docility test Handling test Human approach test Novel human test Novel object test Open field test Social isolation test Temperament score	Activity and exploration behavior in an unknown arena and activity during social isolation Aggressiveness against the handler, running time and number of escapes per minute of test period in presence or absence of the handler Touching the cow from head to tail Behavioral response when a human approaches the cow Behavioral response toward an unknown human Behavioral response toward an unknown object Activity and exploration behavior in an unknown arena Activity and exploration behavior during social isolation Chute score, velocity exit, pen score, flight speed	(50–61)
**(B): GOATS (*****Capra hircus*****)**			
Identity profiles	Behavioral observation	Direct observation during milking and/or over a certain period of time	(21)
Temperament	Behavioral observation Novel human test	Direct observation during milking and/or over a certain period of time Behavioral response toward an unknown human	(62, 63)
Personality	Novel object test Social isolation test	Behavioral response toward an unknown object Call, activity, and exploration behavior during social isolation	(64)
**(C): SHEEP (*****Ovis aries*****)**			
Emotional reactivity	Human approach test Open field test Social isolation test	Behavioral response when a human approaches the sheep Activity and exploration behavior in an unknown arena Activity and exploration behavior during social isolation	(20)
Temperament	Novel object test Open field test Social isolation test	Behavioral response toward an unknown object Activity and exploration behavior in an unknown arena Activity and exploration behavior during social isolation	(65–68)
Personality	Open field test	Activity and exploration behavior in an unknown arena	(69)
**(D): HORSES (*****Equus caballus*****)**			
Coping type	Behavior after reintroduction in a group Novel object test Open field test Owner ratings Water spray test	Expression of submissive or dominant behavior toward conspecifics Behavioral response toward an unknown object Activity and exploration behavior in an unknown arena Questionnaire about the expression of certain behaviors Scoring of the horse's reaction to a spray of water	(70)
Temperament	Behavior after reintroduction in a group Behavioral observation Handling test Horse personality questionnaire (HPQ) Novel human test Novel object test Open field test Owner ratings Social isolation test Surprise test Water spray test	Expression of submissive or dominant behavior toward conspecifics Expression of behaviors in the home pen Latency to allow the human to touch the leg about three times Questionnaire about 43 behaviorally defined adjectives Behavioral response toward an unknown human Behavioral response toward an unknown object Activity and exploration behavior in an unknown arena Questionnaire about the expression of certain behaviors Activity and exploration behavior during social isolation Latency to start eating again after opening an umbrella Scoring of the horse's reaction to a spray of water	(70–75)
Personality	Handling test Horse personality questionnaire (HPQ) Novel human test Novel object test Owner ratings	Latency to allow the human to touch the leg about three times of the horse Questionnaire about 43 behaviorally defined adjectives Behavioral response toward an unknown human Behavioral response toward an unknown object Questionnaire about the expression of certain behaviors	(76–87)
**(E): PIGS (*****Sus scrofa*****)**			
Coping Personality Type	Backtest	number of escape attempts, latency to first escape attempt	(18)
Coping Type	Backtest Behavioral observation Combined open field and novel object test Human approach test Novel object test Resident-intruder test Social support test	Number of escape attempts, latency to first escape attempt Expression of behaviors in the home pen Activity and exploration behavior in an unknown arena with an unknown object Behavioral response when a human approaches the pig Behavioral response toward an unknown object Latency to attack a conspecific, number and duration of fights, lesion score Socio-positive behavior toward a conspecific	(88–96)
Individual differences in behavior	Backtest Combined open field and novel object test Human approach test Novel object test Open door test	Number of escape attempts, latency to first escape attempt Activity and exploration behavior in an unknown arena with an unknown object Behavioral response when a human approaches the pig Behavioral response toward an unknown object Latency to leave the home pen	(25, 97)
Temperament	Human approach test Novel object test Open door test	Behavioral response when a human approaches the pig Behavioral response toward an unknown object Latency to leave the home pen	(98)
Personality	Backtest Open field test	Number of escape attempts, latency to first escape attempt Activity and exploration behavior in an unknown arena	(99)

*The column “Personality-related term used” refers to the term the studies used to explain their results. The column “Test” refers to the behavioral tests used, while the column “Measures” refers to the measured variables*.

### Temperament

In animal personality research, the term temperament is closely related to personality. Temperament often matches the definition found in human personality research and is defined as inherited, early-appearing tendencies that continue throughout life and that serve as the foundation for personality (6) (Table 1). Since the distinction of both terms by definition is often vague, some animal researchers simply treat them as synonyms (29), while others argue that the term temperament is used simply to avoid using the term personality with regard to animals, which might be associated with anthropomorphism by some ethologists (102). While temperament is assumed to be based on early, stable predispositions in the emotional and behavioral responses of an individual like boldness, aggressiveness and pleasantness, personality is often reserved for patterns of reflection and reaction to environmental circumstances acquired during a lifetime in organisms with sophisticated cognitive capacities. The behavioral factors that are the focus of research on animal temperament in many different species are reactivity, fearfulness, sociability, responsiveness, and aggression. Temperament in non-human animals is often described on a one-dimensional scale using expressions such as “proactive vs. reactive,” “aggressive vs. non-aggressive,” “bold vs. shy,” etc. (103, 104). The actual theoretical frameworks on temperament in non-human animals argue for scoring reactions in more than one dimension to reflect the entire nature of temperament (29, 105). A multivariate approach has been used by Meager et al. (106) to investigate whether distinct temperaments are present in Atlantic cod (*Gadus morhua*). Their results refer to the multidimensionality of animal temperament and provide a clear indication that two distinct behavioral phenotypes are evident in fish. A study in non-human primates has shown that temperament, based on a factor analysis including behavior in different dimensions in yearling rhesus monkeys (*Macaca mulatta*), seems to be an important factor in an individual's ability to select friends and is related to later variations in the animals' social networks (107). The subjects preferred peers that had similar temperament scores to their own even after accounting for sex, rank and kinship. This example shows that the definition of temperament from the human domain can be used for non-human animals as well. Foyer et al. (108) investigated the impact of the level of maternal care in dogs on the offspring's behavior at approximately 18 months of age. The authors conducted a set of temperament tests to score the behavior of the dogs in different factors, such as sociability and fearfulness. The study showed that the level of maternal care differed consistently and that it has a significant impact on the adult temperament of the offspring, mainly in terms of sociability and dominance. The responses of cats across different behavioral factors were assessed to calculate a feline temperament profile (109). Later, the reactions of the cats in a 3-min stress test were measured. The authors did not find correlations between temperament scores and measures of behavioral and adrenocortical responses in the stress test. However, many studies concerning individual differences in animal behavior do not make the distinction between temperament and personality consistently, which makes it more difficult to understand the results with regard to temperament and/or personality.

In many farm animal studies, the term temperament is used as an equivalent to the term personality, which also can be seen in Table 2. General responses to unfamiliar humans or situations are quite important for handling, management and selective breeding (65). And traits such as fearfulness, happiness, alertness and docility [(51, 52, 110) can be considered to describe temperament when measured during the juvenile period of an animal and/or once in an animal's life. Animals that are more likely to cope with handling procedures are considered to have a “good” temperament (51) and dairy temperament (generally defined as the animal's response to milking) has been included in the breeding objectives of some countries [reviewed by Gibbons (111)]. Furthermore, high reactive bulls (reactivity was measured with a temperament score) had a greater rectal temperature and a higher cortisol and epinephrine concentration prior to and after transportation than low reactive bulls (53). As these examples measure personality, because measured in adult animals, these examples show how complex it is to describe individual personality in animals and that temperament reflects a sub-aspect of the whole concept of animal personality.

### Coping style

The concept of coping styles has also been used in the past few decades to better understand animal personality and is based on the animal's reaction to its environment with respect to reducing the effect of aversive stimuli (16, 19) (Table 1). Like in humans, animals show individual differences in coping with different environmental changes, confirming that personality and coping style are closely linked [(11, 15, 16, 19, 112); but see Zidar et al. (113) for an example of a missing relationship in the red junglefowl]. According to the boldness-shyness continuum, animals can be categorized into three sub-groups based on their risk-taking behavior: bold, intermediate and shy (112) or proactive, intermediate and reactive based on the animal's reaction to its environment with regard to altering the effect of aversive stimuli (fight/flight response) (11, 15, 16, 19). Proactive individuals are considered to be more aggressive toward conspecifics, show more dominant behavior, and are considered more explorative, bold and active. Moreover, they respond with a strong sympathetic activation and an increase in noradrenergic stimulation when confronted with a challenging situation, while reactive animals are considered the opposite behavioral phenotype, with behavior that is more submissive, less explorative and less active and responding with a strong hypothalamic-pituitary-adrenocortical reactivity toward challenging situations (31, 114–117).

**Figure 2:**
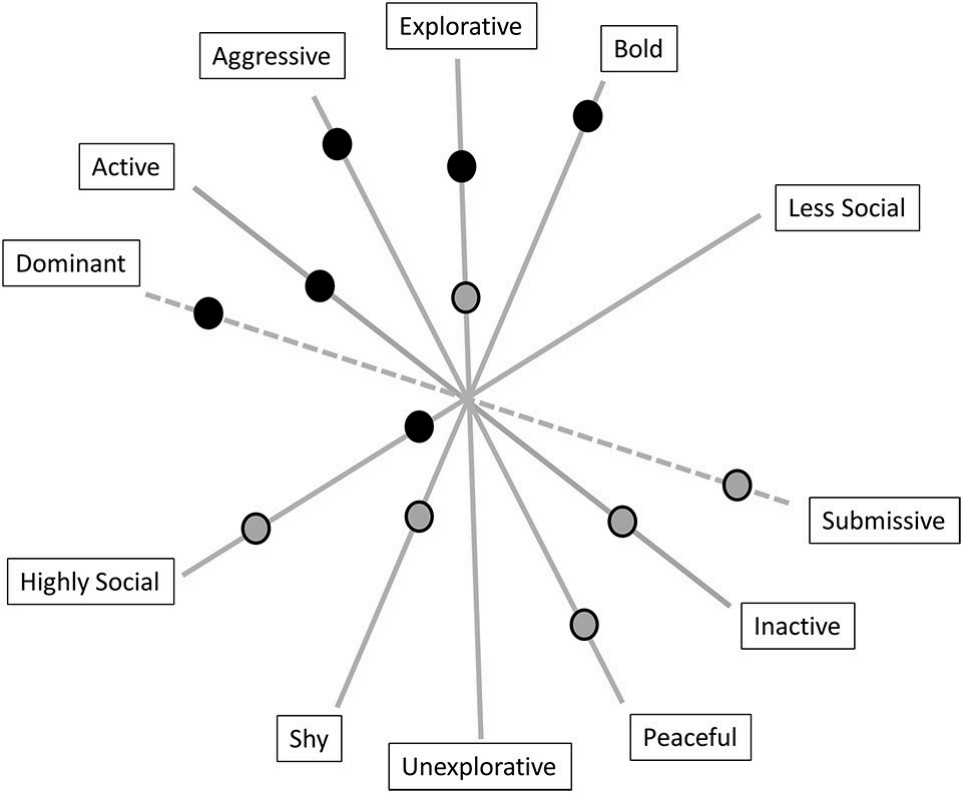
The five basic factors in animal personality research (bold lines) that can also be used to describe coping style. The dominance factor is presented in dashed lines because there are insufficient data to consider it as a fully accepted sixth factor in animal personality. The dots represent two imaginary animals with different coping styles. The animal behaving as described by the black-gray dots would represent a reactive coper, while the animal behaving as described by the black dots would be considered as a proactive individual. Intermediate individuals would express behavior described by a mixture of scores on the factors resulting in individual differences along a continuum.

Figure 2 shows a possible multidimensional approach to describing the five basic factors in animal personality research applied to the concept of animal coping style using an example of two imaginary animals. In addition, there is a potential sixth factor, dominance. There is an ongoing discussion as to whether dominance is a factor by itself or just a social outcome, and it also represents other species-related factors that are often missed in measurements (34, 102). Considering the description of proactive and reactive individuals above, the animal that scores low on the aggressiveness, activity and sociability axes would be described as being more peaceful, inactive and highly social and would represent a reactive coping style. The animal that scores high on the boldness, exploration and aggressiveness axes would be described as being more aggressive, explorative and bold and would be considered as a proactive individual. Intermediate individuals would express behavior described by a mixture of the scores on the factors resulting in individual differences on a continuum. Some factors show only low inter-correlations and are relatively independent from one another, which means that they must not co-occur [reviewed by Uher (118)]. Each factor summarizes the shared variation of diverse inter-correlating traits. The rank-orders of the same individuals on the same factor can vary considerably across different situations. An individual can have different scores on each factor [reviewed by Uher (118)]. The individual-oriented perspective (like in humans and shown in Figure 1) can also be applied to the analyses of multiple individuals such that individuals sharing a similar profile can be grouped statistically into configurational types. Such groups represent distinct and discontinuous categories of prototypes (e.g., proactive, intermediate and reactive) [reviewed by Uher (118)]. According to Koolhaas (119), proactive individuals perform better under highly predictable conditions, while reactive individuals perform better under variable and unpredictable environmental conditions. Proactive individuals also grow faster and have a higher RMR, which they can afford due to their higher rates of food acquisition. When food is abundant, proactive individuals may perform better than reactive individuals [reviewed by Careau et al. (120)]. Many studies have shown that different coping styles correlate with dominance rank, cognitive abilities and physiological measures [e.g., immunology (119)]. In fish, bold female guppies (*Poecilia reticulate*) learned a task more quickly than shy females (121), and in zebra finches (*Taeniopygia guttata*), more active birds were faster at solving a task, while less active individuals needed longer or did not solve the task at all, which indicates that higher activity may lead to routine-forming behavior instead of being attentive to external cues (122). Proactive Senegalese sole (*Solea senegalensis*) juveniles exhibited a shorter feeding latency, a higher duration of escape attempts and a lower cortisol level than reactive individuals (123). An example in mammals revealed that different coping styles in wild alpine marmots (*Marmota marmota*) are accompanied by different baseline and stress-induced plasma oxidative statuses (28). In male rainbowfish (*Melanotaenia duboulayi*), personality factors like aggression, activity and boldness covaried with the male's position in the hierarchy, directly influencing their reproductive success (124). Moreover, an example of spiders living in colonies showed that individuals with different coping styles do different tasks. While aggressive spiders perform prey capture and colony defense, less aggressive spiders perform brood care (22).

Farm animal research on coping styles became more and more important because the coping style covers the aspect of an animal's skill in coping with environmental challenges. Farm animals like pigs (*Sus scrofa*), cattle (*Bos taurus*), and horses (*Equus caballus*) have to cope with numerous challenging situations (e.g., regrouping, housing, management) and technical material during their lives. Therefore, investigating their coping strategies and cognitive abilities appears to be a very important research topic, because like temperament, it reflects sub-aspects of the whole concept of animal personality. Aggression, boldness and exploration seem to be related to results in backtests that determine the specific coping-type in pigs assessed by the early escape behavior of piglets: Proactive pigs proved to be more aggressive, explorative and bolder than reactive pigs (88, 97). Recently, a number of studies were conducted to investigate the impact of different coping styles on psychophysiological measures and molecular correlates later in life. Reactive cows can be distinguished by their baseline HRV (54) and pigs characterized as belonging to different coping styles differed in their general autonomic reaction (e.g., HR, HRV) and in their affective appraisal in relation to common husbandry-related situations like feeding or human handling (125), indicating personality-depended emotional assessment of environmental situations. There is also evidence that individual coping styles of pigs are reflected in their immune responses indicating that proactive pigs might favor molecular pathways enabling a more effective strategy for defense and recovery than in reactive pigs (89, 90). Moreover, a recently conducted genome-wide association study revealed that hypothalamic genes known to be involved in pathways regarding the immune system, telomere function signaling, neurotransmitter receptors as well as circadian rhythms were differently expressed in pigs with different coping styles (126).

## Measures and methods in farm animal personality research

Personality, temperament and/or coping style and how they are interrelated with a diversity of other external or internal stimuli are investigated using different experimental setups. The main problem is to know exactly which personality factor or variable is being investigated using a certain experimental design (e.g., tests of exploration in an open field, test of boldness in investigating a novel object). Murphy et al. (127) identify in their review the most important aspects of experiments used to investigate farm animal personality. A test should be ecologically valid and the animal should be able to display its natural emotion-related behaviors. Since emotions have recently been discussed to be a personality factor by itself [see below reviewed by Koolhaas et al. (128)], tests should be sensitive enough to capture slight differences in levels of emotional arousal (bodily activation or excitation, the first dimension of emotion) or valence (negative or positive, the second dimension of emotion) (129, 130), which is important for longitudinal studies. Furthermore, the test itself and the variables measured should be standardized to allow for comparisons between studies (127). In his review, Gosling (3) describes the variables that can be used to analyse personality factors: (i) reactivity, emotionality, fearfulness: e.g., measurement of the defecation rate in an open field; (ii) exploration/boldness: e.g., interactions with a novel object; (iii) sociability: e.g., frequency of social encounters; (iv) aggression: e.g., latency to attack another individual; (v) activity: e.g., amount of enclosures (e.g., open field or arena test) covered; (vi) dominance/assertiveness: e.g., the individual's rank in the dominance hierarchy. Of course, these variables can be used to analyse other personality factors as well, and the mentioned personality factors can be measured by other variables or even a mixture thereof.

The most important aspect is to find the most appropriate test with the most appropriate variables to record the behaviors that are desired to analyse the intended personality factors for each species. Different species have different environmental requirements because they have different habitats and different life-histories. Koski (34) indicated that most studies about personality focus on boldness, aggressiveness, activity, exploration and sociability as the most prominent personality factors. However, she writes that the focus on these personality factors derived from human personality research may ignore the possibility of other factors that are more important for the investigated species and that such a focus limits our understanding of the full repertoire of personality factors [also reviewed by Gosling and John (32)]. Therefore, it is possible that species not only differ in the type of relevant factors, but also in the number of such factors (34): A study done on 1223 horses of eight different breeds with a Horse Personality Questionnaire also identified approximately six personality factors (78). Carter et al. (131) found boldness to be one of the most commonly measured personality factors in animal personality research, and it has been interpreted as the propensity to take risks, especially in novel situations, and as an individual's response to a risky situation faced alone. Boldness is often tested by quantifying behavioral responses to novel objects, responses to a novel environment, and/or responses to predation risk. However, these three types of tests are not necessarily comparable and demonstrate a lack of standardization for quantifying this behavior. For example, measuring boldness in cavies is slightly different than measuring boldness in pigs and cattle. For the three species, one can use the novel object test. While in cavies, the novel object test is conducted in the home enclosure (30, 47, 48), with pigs and cattle, the test is normally conducted in an arena outside the home pen after a habituation period or after an open field test [reviewed by Forkman et al. (132)]. Tests like the novel human test or the open-door test are conducted with farm animal species and measure the reactivity of an individual toward unknown persons or places (55, 71, 98). This type of test is only feasible with domesticated species that are used to human manipulation such as farm animals. In most cases, the measured trait is suggested to mirror fearfulness when describing an animal having a higher latency in approaching an unknown person [reviewed by Murphy et al. (127)]. In rodents, tests like the elevated plus maze [e.g., in mice and rats (133, 134)] or leaving a plastic shelter in an open field [e.g., in cavies (47)] are used to measure fearfulness or its opposite, fearlessness. Exploration is measured in most mammal studies and in most farm animal species with an open field test [reviewed by Forkman et al. (132), but see recent critiques by Perals et al. (135)].

To measure different coping strategies in pigs, Hessing et al. (136) suggested conducting the backtest at an early age to measure defensive reactions like struggling while the piglet is turned over on its back. Zebunke et al. (91) used more than 3,000 individuals and repeated the test four times with each individual. The results showed a moderate consistency of behavioral reactions across repeated testing, which cannot be attributed to a randomly occurring pattern. The authors concluded that the backtest does not provide phenotypic evidence for definitive coping styles that are clearly separable. Instead, they found pronounced individual dispositions along a continuum from proactive to reactive coping behaviors. The results of an equivalent to the backtest conducted with juvenile cavies before weaning (the struggle test) show a similar pattern: the time spent struggling was not significantly repeatable but showed a trend (137). Therefore, the backtest may indicate a certain coping style in pigs, which is discussed to be a personality factor by itself (113, 128). The backtest should not be used as a reference on its own but rather should be used as an addition to other tests measuring behavior and a certain personality type. Moreover, these examples confirm that one test can simultaneously be influenced by and therefore measure two or more personality factors (29).

**Figure 3:**
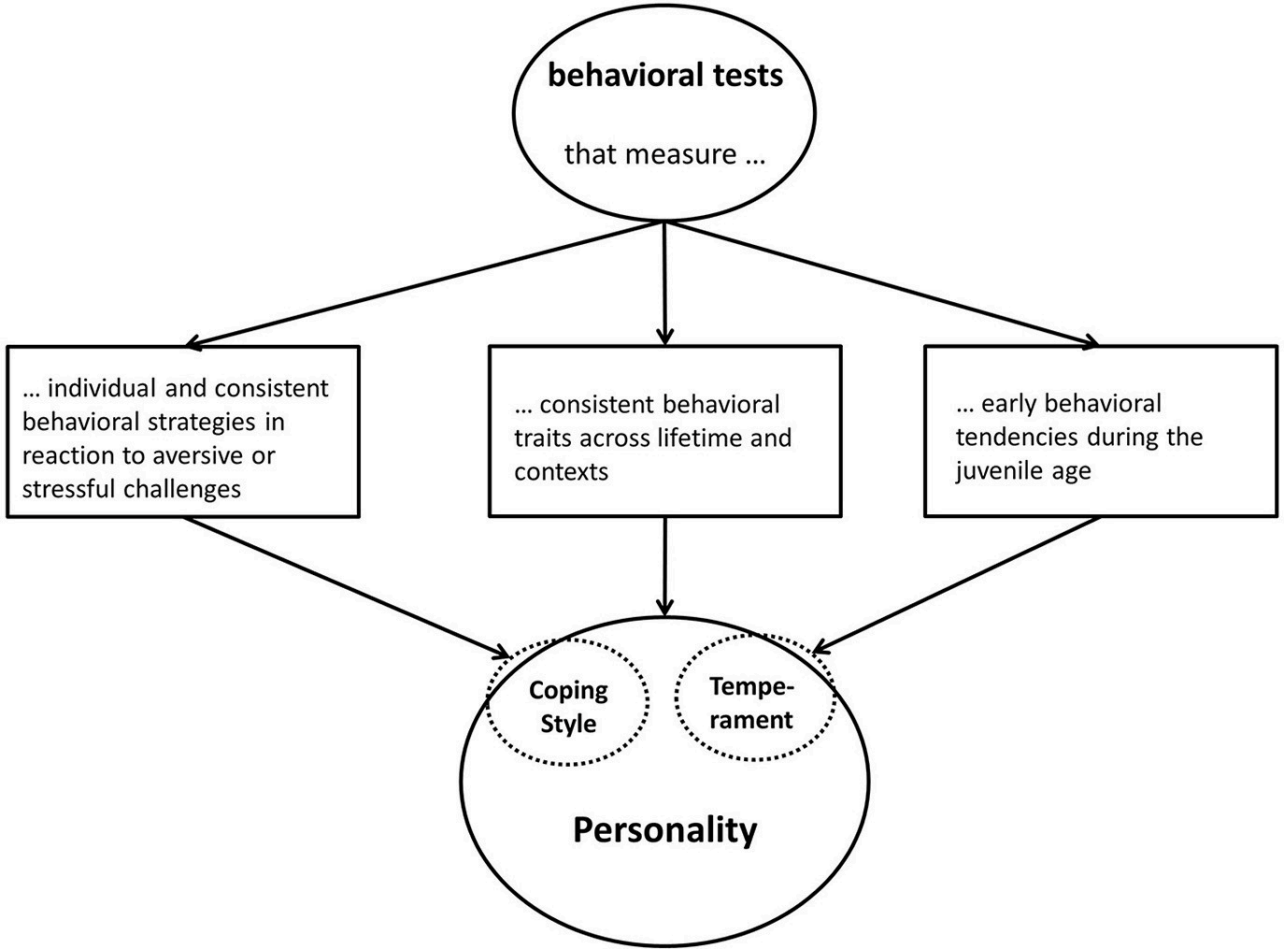
Various behavioral tests that can be used to assess different aspects of personality. It becomes evident that coping style and temperament are sub-aspects of the concept of personality.

Besides the fear of anthropomorphism in using the term personality, to know what is measured is one of the biggest lacking points in the field of farm animal personality research (see Table 2). Inspired by the definitions in Table 1, Figure 3 shows how researchers could proceed to know which personality related term they should use for their interpretation. Temperament and coping style overlap with personality, because they are sub-aspects of the whole personality concept. With all the behavioral tests used in farm animal personality research (e.g., open field test, novel object test, open door test, backtest etc.) applied ethologist can measure different aspects of personality: It depends of the approach they use. For instance, (i) using a behavioral test to measure a response to an aversive or stressful stimulus or situation (e.g., approach or avoidance) means that primarily a specific coping style is assessed, (ii) conducting various behavioral tests in a combination and/or conducted at least twice during lifetime, measures cross-context correlations and/or consistency and therefor assesses personality, (iii) conducting behavioral tests during the juvenile stage and only once during lifetime, would assess temperament.

## Personality and welfare in farm animals

**Figure 4:**
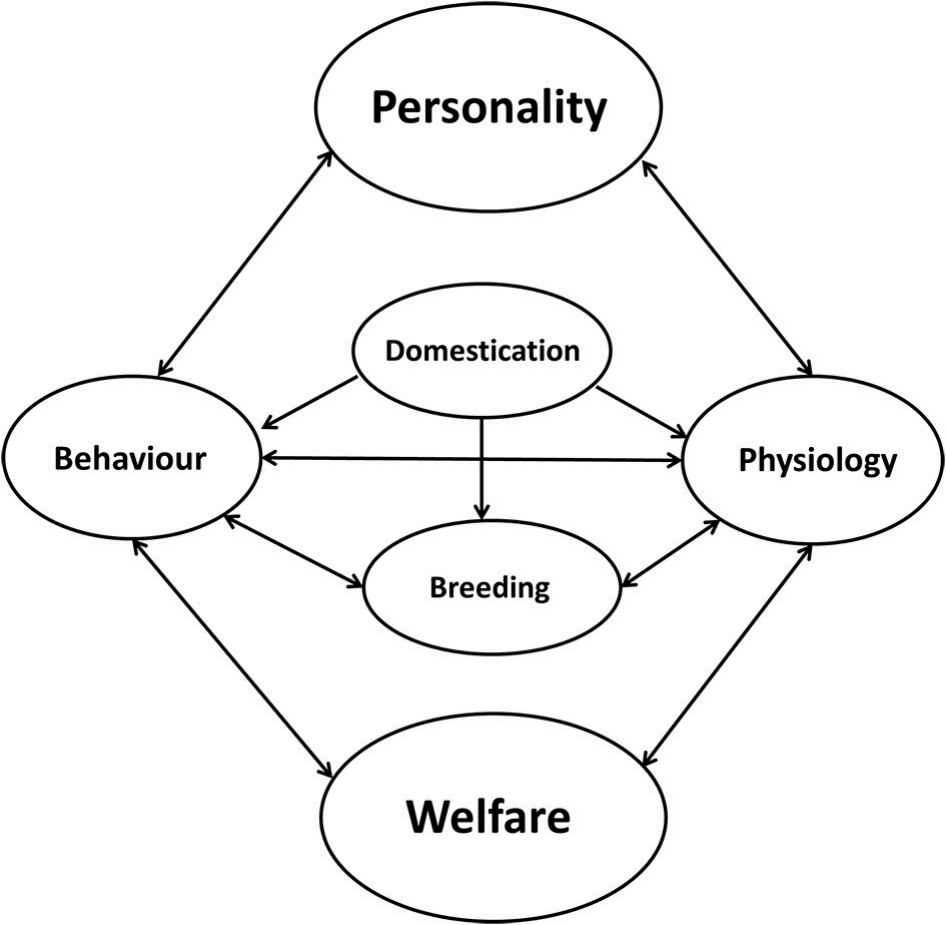
Relationship between personality and welfare. Personality directly influences behavior and physiology and therefore influences individual welfare, while as in a feedback-loop, welfare can directly influence behavior and physiology. Behavior can influence physiology and *vice versa* in a sort of positive feedback system. Especially in farm animals, domestication has an impact on behavior and physiology and directly influences breeding.

In human psychology, studies show that subjective well-being is associated with health (138) and is linked to personality [reviewed by Weiss et al. (139)]. Well-being correlates with the five-factor model of personality, especially with neuroticism, extraversion and conscientiousness (140). In zoo animals, studies indicate that zoo keepers are able to reliably rate animal personality traits and that those ratings are implemented into zoo management practices to improve the welfare of captive animals (141). Personality research in farm animals has become important as well because welfare not only comprises the actual health status of an animal but also is affected by individual differences in behavior and physiology (142). Dawkins (143) already stated that individual behavior has a big advantage in welfare studies because it might become an “early warning system” of trouble yet to come. Changes in behavior (e.g., aggressiveness toward the caretaker) can be a hint of pain or other problems (144). Therefore, investigating and understanding individual differences in behavior, respective to personality, in farm animals is a possible means of measuring states of welfare and can also help to increase welfare. Individuals that are less well adapted to their environment may have reduced welfare, which in turn can lead to reduced productivity (145, 146). Figure 4 represents the influence of personality on individual welfare. Personality directly influences behavior and physiology and therefore influences individual welfare, while as in a feedback-loop, welfare can directly influence behavior and physiology. Behaviors can influence physiology and *vice versa* in a sort of positive feedback system as well. Especially in farm animals, domestication has an impact on behavior and physiology and directly influences breeding. During domestication of most farm animal species, behavior changed to lower levels of aggression and activity (147). Artificial selection for the improvement of production traits may have resulted in the selection of animals that would count as reactive copers (147). Genetic studies of captive animals often rely on the selection of specific production traits (148–150), because specific production traits are emphasized in the individual pedigree of an animal used for breeding [(151, 152); reviewed by Laine and van Oers (153)]. Recently, personality traits have been considered to a greater extent in the calculation of breeding indices because some of these traits show moderate to high heritability (150, 154–156). Aggression is highly heritable in pigs [ranging from h^2^ = 0.32 to h^2^ = 0.48 (157–159)]. In pigs as well as in cattle, aggressive behavior toward stockpersons and group-members is related to increased maternal behavior, which can be problematic [reviewed by Haskell et al. (155, 158)]. In beef and dairy cattle, handling shows moderate to high heritability scores: e.g., chute score (h^2^ = 0.24), flight speed (h^2^ = 0.36), and docility (h^2^ = 0.26) [reviewed by Haskell et al. (56, 155, 160)]. Especially in dairy cattle, milking temperament shows a moderate heritability (e.g., on average h^2^ = 0.19), but is also related to production traits such as milk yield [reviewed by Haskell et al. (56, 155)]. In France the docility test has been used to select for improved temperament in Limousin cattle since 1992, and dairy temperament (generally defined as the animal's response to milking) and milking speed have been included in the breeding objectives of some countries [e.g., United Kingdom and Norway reviewed by Gibbons (111)]. Horses, especially stallions considered for breeding purposes, are judged for performance (e.g., the gaits under the rider, jumping ability, rideability, fitness, health, stamina) and personality (e.g., behavior during handling (labeled as character), attention and reactivity (labeled as temperament) and braveness, willingness and ability to learn [labeled as willingness to work (72)].

Another approach to explain the relationship between personality and welfare has been reviewed by Koolhaas and van Reenen (128), and it describes a three-dimensional model with coping style, emotionality and sociability as independent factors. These factors are defined as being stable over time and across contexts within the individual. Emotionality seems to be one important aspect to increasing welfare because it makes the distinction between fearful animals that are highly emotionally aroused by a challenging situation and non-fearful animals that do not perceive the same situation as stressful or alarming (128). A highly emotionally aroused animal would therefore exhibit activation of neuroendocrine systems while a non-fearful animal do not show any enhanced biological responses (128).

Some studies have shown evidence that emotionality, and therefore personality, seem to have an impact on production traits, such as meat quality and milk production. Studies on steers show that individual differences in stress responses (e.g., increase of cortisol in a stressful situation) or in blood lactate levels have an impact on meat tenderness (57, 58). In dairy cows, personality has an impact on behavioral and physiological responses to milking and on the stress associated with being milked in a novel environment (50, 161). A study on horses shows that personality has an influence on pain expression. Horses that were highly affected by vertebral problems showed more aggressive behavior toward humans than horses with no vertebral problems (144). Lameness was more expressed by highly extraverted horses even if the severity level of the injury threshold was lower when compared to more neurotic individuals (76). A study on female sheep (*Ovis aries*) showed that animals typed as “nervous” [measurement of the behavioral reactivity to the psychosocial stress of social isolation and selected for “calm” or “nervous” temperament for 17 generations (66)] seem to show a decrease in agitation score, the frequency of vocalizations and the plasma concentrations of cortisol when treated with lavender oil as an alternative treatment to alleviate anxiety compared to the response of “calm” sheep (66). Moreover, these “nervous” sheep produced a lower volume of higher viscosity colostrum than “calm” sheep, and this disparity could be corrected by nutritional supplementation (with barley), which only had an effect on “nervous” sheep (162). These examples indicate that personality seems to be an important factor in the efficacy of certain substances and nutritional supplementations. Personalized medicine in animals and humans already indicates that personality is a strong indicator for pathology development, medical treatment and substance efficacy (163). Understanding the individual personalities of farm animals is not only important for their welfare but also has an impact on economic factors for farmers [reviewed by Clark et al. (164)]. Therefore, it is important to think about considering to draw an individual personality profile like in Figure 1 [adapted from Costa and McCrae (33)] to picture farm animal personality and in concordance to improve management, handling, breeding, medical treatment and the design of housing systems that allow the animal to perform effective coping behavior [reviewed by Wechsler (11)].

## Conclusion

This review gives an impression of how diverse farm animal personality research is and which aspects have to be considered in investigating personality in different farm animal species. Terminology in farm animal personality research is somewhat confusing and in some cases difficult to compare because the terms used seem somehow to be species-related. While studies in different mammals, birds, and other taxa widely use the term personality to describe between-individual consistency in behavioral variation, in farm animal research the terms temperament and coping style are predominantly used, probably because personality might be associated with anthropomorphism. The broad field of personality research generally needs more consistency in using theoretical concepts, terms and measures and we recommend that the terms should neither be regarded as synonyms nor as independent terms for consistent behavioral responses in animals, but as different aspects of the whole personality concept. Research on personality of farm animals is currently far from covering all possible aspects, but focuses in particular on the phenotyping of personality traits and potential relationships with cognition, emotion and welfare. We conclude that the assessment of personality in farm animals is of growing scientific, practical and economic interest, because it has an obvious verifiable impact on the individual behavioral reaction to different housing systems, management practices and veterinary interventions and is therefore important for the improvement of animal welfare.

## Author contributions

M-AF, JL, and BP contributed to the conception and structure of the manuscript. Main parts of the manuscript were written by M-AF. The final version of the manuscript was prepared and edited by M-AF, JL, and BP.

### Conflict of interest statement

The authors declare that the research was conducted in the absence of any commercial or financial relationships that could be construed as a potential conflict of interest.
